# A stable association with PME‐1 may be dispensable for PP2A demethylation – implications for the detection of PP2A methylation and immunoprecipitation

**DOI:** 10.1002/2211-5463.12485

**Published:** 2018-08-01

**Authors:** Ryotaro Yabe, Shunya Tsuji, Satoru Mochida, Tsuyoshi Ikehara, Tatsuya Usui, Takashi Ohama, Koichi Sato

**Affiliations:** ^1^ Laboratory of Veterinary Pharmacology Joint Faculty of Veterinary Medicine Yamaguchi University Japan; ^2^ Priority Organization for Innovation and Excellence Kumamoto University Japan; ^3^ Department of Food Science and Technology National Fisheries University Shimonoseki Japan; ^4^ Laboratory of Veterinary Pharmacology Faculty of Agriculture Tokyo University of Agriculture and Technology Fuchu Japan

**Keywords:** demethylation, protein methylation, protein phosphatase 2A, protein phosphatase methyl‐esterase

## Abstract

Reversible methyl‐esterification (methylation) of Leu309 in the protein phosphatase 2A catalytic subunit (PP2Ac) is essential for proper biogenesis of the PP2A holoenzyme. Accumulating evidence links PP2Ac methylation to diseases, including cancer and neurodegenerative disorders. Protein phosphatase methyl‐esterase (PME‐1) specifically catalyzes PP2Ac demethylation. We demonstrate that PP2Ac is demethylated in cell extracts even at 0 °C unless prevented by a PME‐1 methyl‐esterase inhibitor. This promotes dissociation of PP2A heterotrimers with B55 or PR72 subunits, but not those with B56 subunits. These results reveal differential sensitivity of ABC heterotrimers to methylation status of the C subunit. Our study advocates caution when interpreting earlier findings, offers an effective protocol for preserving PP2A complexes, and reveals key distinctions between B subunits and their interactions with the AC core dimer of PP2A.

**Abbreviations**
OA: okadaic acid
PME‐1: protein phosphatase methyl‐esterase
PP2A: protein phosphatase 2A

Protein phosphatase 2A (PP2A) is a major serine/threonine protein phosphatase that regulates multiple cellular processes 1. Deregulation of PP2A has been linked to many diseases, such as cancer 2, 3, 4 and neurodegenerative disorders 5, 6. The PP2A core enzyme consists of a 36‐kDa catalytic C subunit (PP2Ac) and a 65‐kDa scaffolding A subunit. This core enzyme is associated with one of the regulatory B subunits to form the PP2A trimer. The B subunits have four different families, namely B (B55 or PR55), Bʹ (B56 or PR61), Bʹʹ (PR48, PR72, and PR130), and Bʹʹʹ (PR93/PR110), and control the localization and substrate specificity of PP2A 1, 7.

The reversible methylation of the C‐terminal Leu309 residue of PP2Ac is an essential regulatory mechanism for the proper biogenesis of PP2A holoenzyme 8. Methylation of PP2Ac has been shown to enhance the affinity of the PP2A core enzyme for a subset of the regulatory subunits in cells 9, 10. Methylation/demethylation of PP2Ac is catalyzed by the leucine carboxyl methyltransferase and protein phosphatase methyl‐esterase (PME‐1), respectively 11, 12. We have previously reported that the loss of PME‐1 enhances the association of B55 with PP2A core enzyme 13. In addition to its role as a methyl‐esterase, PME‐1 is suggested to work as PP2A inhibitory protein by directly binding to the active site of PP2Ac 14. Accumulating evidence has revealed that the deregulation of PP2Ac methylation has implications in cancer and Alzheimer's disease 15, 16, 17, 18, 19. Therefore, an assay system to analyze PP2A methylation levels would be valuable. Here, we use a reliable immunoblotting assay and *in vitro* alkaline hydrolysis to quantitate the fraction of methylated PP2A. The PP2A inhibitor, okadaic acid (OA), is widely used as an additive in assay buffer to prevent PP2Ac demethylation, presumably by blocking PME‐1 from binding to the PP2Ac active site 13, 20, 21. However, little attention has been paid to understand whether the inhibition of PME‐1/PP2Ac association is sufficient to prevent PP2Ac demethylation.

## Materials and methods

### Animals and cell cultures

Mouse embryonic fibroblasts (MEFs) were isolated from embryos (ED: 12.5–14.5) of wild‐type (WT) and PME‐1 knockout (KO) mice as previously described 13, and were grown in Dulbecco's modified Eagle's medium (DMEM) containing 10% FBS and 1× antibiotic/antimycotic (Thermo Fisher Scientific, Waltham, MA, USA). Experimental protocols were approved by Yamaguchi University Animal Care and Use Committee. HEK293T cells (Takara Bio, Shiga, Japan) were grown in DMEM containing 10% FBS and 1× antibiotic/antimycotic.

### Antibodies

Antibodies were obtained from the indicated supplier: anti‐PME‐1 (sc‐25278; Santa Cruz Biotechnologies, Dallas, TX, USA), anti‐demethyl PP2Ac (Clone 4B7, sc‐13601; Santa Cruz Biotechnologies), anti‐FLAG (F7425; Sigma‐Aldrich, St. Louis, MO, USA), anti‐total PP2Ac (07–324; Merck Millipore, Burlington, MA, USA), and p97/valosin‐containing protein (VCP; GTX113030; GeneTex, Hsinchu‐City, Taiwan).

### Plasmid construction and lentivirus production

3 × FLAG‐Human PME‐1 WT and S156A, PP2A B55α, and B56α were previously described 13. Human PME‐1 M335D was generated using In‐Fusion HD Cloning Kit (Takara Bio), with pLVSIN 3 × FLAG‐PME‐1 WT as the template. 3 × FLAG‐tagged human PP2A B56γ2, B56γ3, and PR72 were PCR amplified from human liver cDNA and subcloned into pLVSIN‐EF1α‐IRES‐ZsGreen1 vector. pGEX1‐PME‐1 WT, S156A, M335D, pHEX1‐PME‐1 WT, S156A, and M335D were generated using In‐Fusion HD Cloning Kit, with pLVSIN 3 × FLAG‐PME‐1 plasmids as the template. To produce lentiviruses, 3 μg of pLVSIN, 2.3 μg of a packaging plasmid (psPAX2), and 1.3 μg of a coat‐protein plasmid expressing vesicular stomatitis virus G protein (pMD2.G) were transfected into Lenti‐X 293T cells (Takara Bio) cultured in 60‐mm dishes using PEI Max (Polysciences, Warrington, PA, USA) according to the manufacturer's instruction. Viral supernatants were collected after 48 h and, after filtering (0.22 μm), were added to cells for 8 h 13.

### Immortalization of MEF

EGIP‐EF1a‐Large T‐IRES‐Puro, a lentiviral plasmid expressing simian virus 40 large T antigen (LT) but not small T antigen (ST), was obtained from Addgene (Cambridge, MA, USA) (ID18922). Primary MEFs were infected with virus for 8 h, and with puromycin treatment for 6 days, immortalized LT‐positive cells were selected.

### Immunoblotting

The cells and tissue samples were lysed in a ‘standard lysate buffer’ containing 50 mm Tris/HCl (pH 8.0), 5 mm EDTA, 5 mm EGTA, 1% Triton X‐100, 1 mm Na_3_VO_4_, 20 mm sodium pyrophosphate, and Roche Complete protease inhibitor mixture. For measuring PP2Ac methylation level, 1 μm of ABL127 (PME‐1 inhibitor; Sigma‐Aldrich) was added in a lysate buffer. For tissue samples, Multi‐Beads Shocker (Yasui Kikai, Osaka, Japan) was used to homogenate tissues according to manufacturer's instruction.

Immunoblotting was performed as previously described 22. Proteins were separated by SDS/PAGE and transferred onto nitrocellulose membrane (FUJIFILM Wako, Osaka, Japan). The membranes were blocked with 0.5% skim milk and treated with primary antibodies, and immunoreactive bands were visualized using ECL Pro (PerkinElmer, Waltham, MA, USA) and LAS‐3000 (GE Healthcare, Chicago, IL, USA). Band densities were quantified using imagej densitometry analysis software (National Institutes of Health, Bethesda, MD, USA). p97/VCP was used as a loading control.

### Immunoprecipitation

Immunoprecipitation was performed as previously described 13. Cells expressing 3 × FLAG‐tagged proteins were lysed in a buffer containing 40 mm HEPES, 150 mm NaCl, 2 mm EDTA, 10 mm disodium glycerophosphate, 0.3% CHAPS, 10 mm sodium pyrophosphate, and Roche Complete protease inhibitor, sonicated 4 times for 5 s each time, then the lysates were centrifuged at 15 000 ***g*** for 15 min. Supernatants were incubated with FLAG M2 beads (Thermo Fisher Scientific) at 4 °C for 2 h. Beads were washed three times with lysis buffer. Bound proteins were eluted with SDS sample buffer and subjected to immunoblotting.

### Detection of PP2A methylation

Protein phosphatase 2A methylation level was analyzed indirectly using anti‐demethylated PP2Ac antibody as previously described 13. Briefly, a cell extract sample was divided into several tubes, and one of them was treated with 1 m NaOH to remove methyl groups. Samples were subjected to immunoblotting with anti‐demethylated PP2Ac antibody. Bands of NaOH‐treated samples represent total PP2Ac protein levels, and the level of methylated PP2Ac was calculated as the ratio of band densities normalized with NaOH‐treated samples as 100%.

### Purification of recombinant protein

pGEX1‐PME‐1 WT, S156A, M335D, pHEX1‐PME‐1 WT, S156A, M335D were transformed into competent cells of *Escherichia coli* strain BL21 DE3 Codon Plus RP (Agilent Technologies, Santa Clara, CA, USA). Cultured medium was amplified to larger volume in 200 mL of LB medium containing 100 μg·mL^−1^ ampicillin and 35 μg·mL^−1^ chloramphenicol in incubator at 37 °C. Protein expression of GST‐PME‐1 and His‐PME‐1 were induced with 0.2 mm IPTG at 26 °C for 3 h, 1 mm IPTG at 37 °C for 1 h, respectively. Cultured cells were harvested at 2150 ***g*** for 15 min at 4 °C, and pellet was resuspended in recombinant buffer A (20 mm Tris/HCl pH 7.5, 105 mm NaCl) and extracted with buffer B (20 mm Tris/HCl pH 7.5, 105 mm NaCl, 0.1 mm EGTA, 5 mm benzamidine, 1 mm PMSF, 0.1% Tween‐20) with lysozyme and disrupted by sonication. Supernatant fraction collected by centrifugation at 13 200 ***g*** 10 min was purified with Glutathione Sepharose beads (GE Healthcare) and HisPur Ni‐NTA (Thermo Fisher Scientific). Purified protein extraction buffer was replaced to recombinant storage buffer (20 mm Tris/HCl pH 7.5, 150 mm NaCl, 0.01% Tween‐20) using PD‐10 desalting column (GE Healthcare) and quantified by comparing with BSA protein on Coomassie Brilliant Blue‐stained gels.

Recombinant His‐PP2Ac was synthesized in insect cells High Five (Thermo Fisher Scientific) by infection of recombinant baculoviruses encoding His × 8‐tagged human PP2Ac using a baculovirus expression system and purified as described previously 23, 24.

### His pull‐down assay

Cells were lysed in an EDTA‐free CHAPS buffer (40 mm HEPES, 150 mm NaCl, 2 mm EDTA, 10 mm disodium glycerophosphate, 0.3% CHAPS, 10 mm sodium pyrophosphate, and Roche Complete protease inhibitor). Equal amount of cell extracts were dispensed to 1.5‐mL tube and incubated with recombinant PME‐1 and HisPur Ni‐NTA resin at 4 °C for 2 h with gentle agitation. After centrifugation, the pellets were washed three times with lysate buffer and subjected to SDS/PAGE analysis.

### GST pull‐down assay

Recombinant His‐PP2Ac and GST‐PME‐1 were incubated with Glutathione Sepharose beads in recombinant buffer A with indicated reagents at 4 °C for 2 h with gentle agitation. After centrifugation, the pellets were washed three times with recombinant buffer A with indicated reagent and subjected to SDS/PAGE analysis.

### *In vitro* PP2A activity assay

Recombinant His‐PP2Ac and His‐PME‐1 were incubated in 96‐well plate with phosphatase assay buffer (50 mm Tris, pH 7.5, 10 mm MgCl_2_, Brij‐35 0.02%, pH 7.5) for 3 h. After incubation, phospho‐peptide substrate (K‐R‐pT‐I‐R‐R) was added and free phosphate is detected by malachite green and molybdic acid as previously described 13. PP2A activity is determined by measuring 620‐nm absorbance and comparing to a phosphate standard curve.

### Statistical analysis

The results are expressed as the means ± SE. Comparisons between the groups were performed by one‐way analysis of variance, followed by Student–Newman–Keuls test. For all analyses, a probability value of *P* < 0.05 was considered statistically significant.

## Results

### PP2Ac is demethylated in the cell extracts in a time‐dependent manner

**Figure 1:**
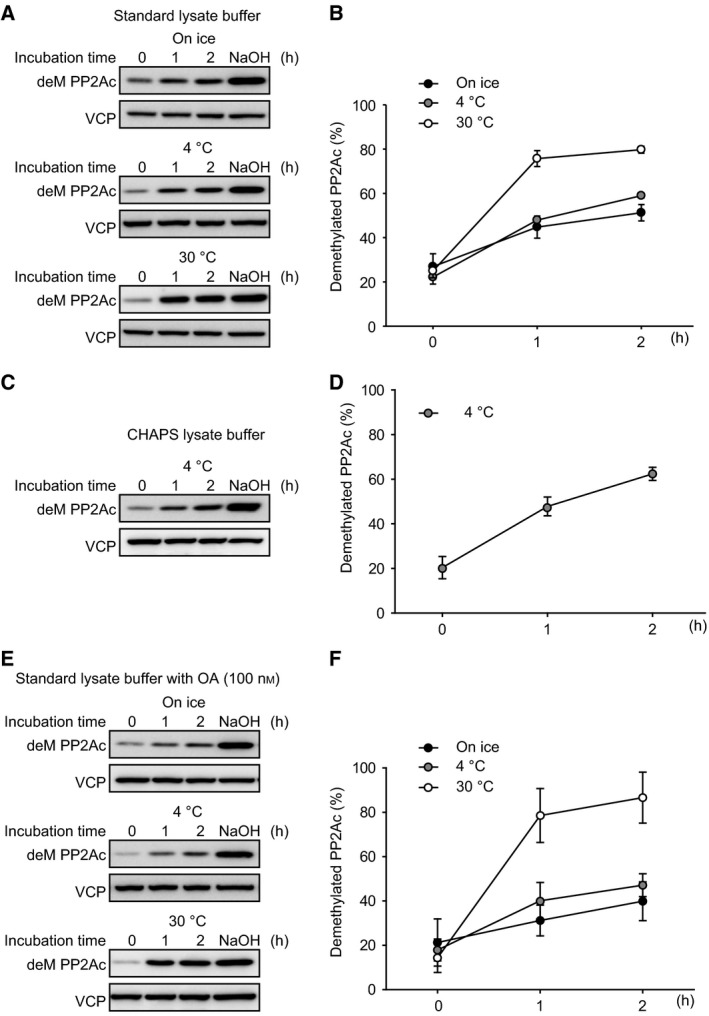
PP2Ac is demethylated in the cell extracts in a time‐dependent manner. HEK293T cells were lysed in a standard buffer (A,B), CHAPS buffer (C,D), and a standard buffer with OA (100 nm) (E,F). The cell extracts were incubated on ice, at 4, and 30 °C for indicated time periods, and then treated with or without NaOH (0.1 m). Demethylation of PP2Ac was detected by immunoblotting with anti‐demethylated PP2Ac antibody (deM PP2Ac), and the ratio of deM PP2Ac was determined by NaOH‐treated cell extracts as 100%. Representative images (A,C,E) and quantitative data (B,D,F) from three independent experiments are shown. The results are expressed as the means ± SE. VCP was used as a loading control.

Methylation of PP2Ac has been reported to affect its association with a subset of the B subunits 9, 10, but there have not been any studies that analyzed the time‐dependent change of PP2A methylation levels in cell extract. Here, we examined different incubation parameters relative to PP2A methylation levels. We lysed HEK293T cells with Tris/HCl/Triton X lysate buffer (standard lysate buffer), and the cell extracts were incubated for 2 h on ice, at 4 °C, or at 30 °C. Incubation of the cell extracts promoted demethylation of PP2Ac in a time‐dependent manner, even on ice (Fig. 1A,B). The rate of hydrolysis was about the same at 4 and 0 °C, which was substantially slower than at 30 °C. Even when chilled the fraction of demethylated PP2Ac more than doubled in < 2 h. Demethylation of PP2Ac during the incubation of the cell extracts also was observed with CHAPS buffer, which is widely used for immunoprecipitation (Fig. 1C,D). OA, a PP2A inhibitor, has been reported to prevent PP2Ac demethylation by blocking the PME‐1 binding to the PP2Ac active site 12, 14. Based on these reports, we added 100 nm OA to the standard lysate buffer and assayed PP2Ac demethylation *in vitro*. Interestingly, we found that OA had essentially no effect on PP2Ac demethylation at 30 °C and only a minor effect at lower temperatures (Fig. 1E,F). These results indicate that addition of OA is not enough to effectively inhibit PP2Ac demethylation in cell extracts.

### Inhibition of PME‐1 methyl‐esterase activity blocks PP2Ac demethylation in the cell extracts

**Figure 2:**
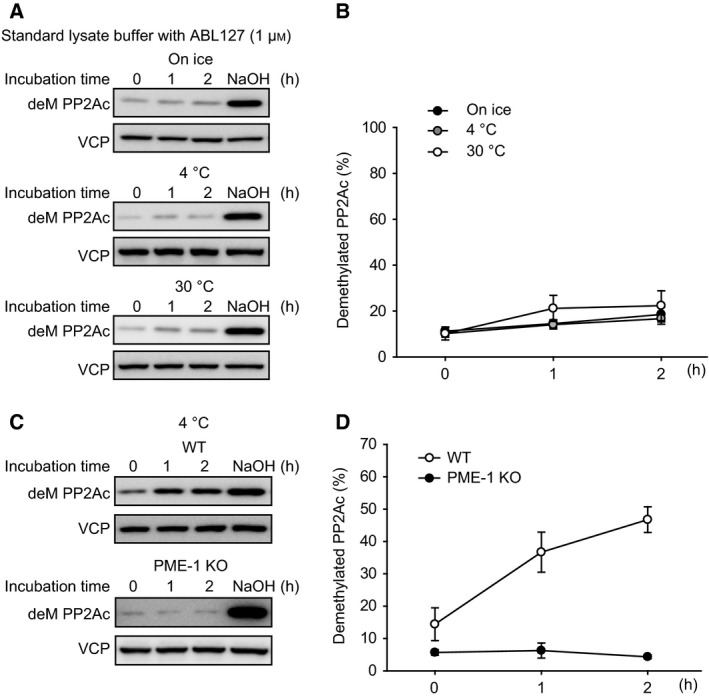
Inhibition of PME‐1 methyl‐esterase activity blocks PP2Ac demethylation in the cell extracts. (A,B) HEK293T cells were lysed in a standard buffer with PME‐1 methyl‐esterase inhibitor ABL127 (1 μm), and demethylation of PP2Ac was detected by immunoblotting with anti‐demethylated PP2Ac antibody (deM PP2Ac). The cell extracts were incubated on ice, at 4, and 30 °C for indicated time periods, and then treated with or without NaOH (0.1 m). The ratio of deM PP2Ac was determined by NaOH‐treated cell extracts as 100%. Representative images (A) and quantitative data (B) from three independent experiments are shown. The results are expressed as the means ± SE. (C,D) WT MEFs and PME‐1 KO MEFs were lysed in a standard buffer. The cell extracts were incubated at 4 °C for indicated time periods. Demethylation of PP2Ac was detected by immunoblotting with anti‐demethylated PP2Ac antibody (deM PP2Ac), and the ratio of deM PP2Ac was determined by NaOH‐treated cell extracts as 100%. Representative images (C) and quantitative data (D) are shown. *N* = 3 (WT) and 5 (KO). The results are expressed as the means ± SE.

As another approach to prevent demethylation of PP2Ac in cell extracts, we analyzed the effects of the PME‐1 methyl‐esterase inhibitor ABL127. We added ABL127 (1 μm) to the standard lysate buffer and measured the methylation level of PP2Ac in extracts of HEK293T cells. We observed two effects: First, the initial level of PP2Ac methylation was about half as much relative to extracts without ABL127; and second, PP2Ac demethylation was effectively and completely inhibited (Fig. 2A,B). To test whether this PP2Ac demethylation was catalyzed by PME‐1, we compared extracts of WT and PME‐1 KO MEFs. We observed time‐dependent PP2Ac demethylation in extracts of WT MEFs, as expected, while in contrast there was no demethylation of PP2Ac in extracts of PME‐1 KO MEFs (Fig. 2C,D). These data confirmed that PME‐1 is the sole enzyme involved in PP2Ac demethylation in the cell extracts.

### PME‐1 M335D mutant has reduced binding to PP2Ac

**Figure 3:**
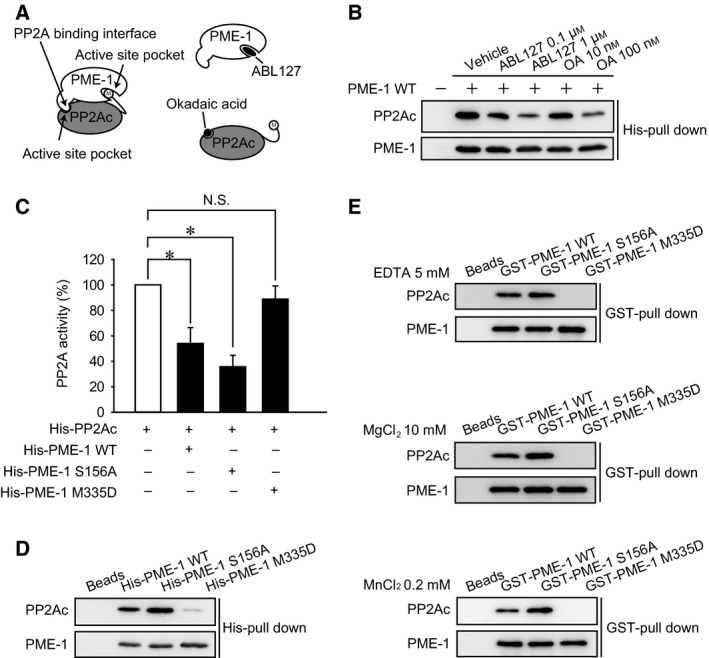
PME‐1 M335D mutant has reduced binding to PP2Ac. (A) Schematic image of PME‐1/PP2Ac association. (B) Recombinant His‐PME‐1 WT was incubated with PME‐1 KO MEFs cell extracts with DMSO, ABL127 (0.1 and 1 μm), or OA (10 and 100 nm). His‐PME‐1 was pulled down with Ni‐NTA agarose, and the association of His‐PME‐1 with endogenous PP2Ac was analyzed by immunoblotting. Representative images from three independent experiments are shown. (C) Recombinant His‐PME‐1 WT, S156A, and M335D mutants were incubated with recombinant PP2Ac, and PP2A activity was determined by *in vitro* PP2A activity assay. The percentage of PP2A activity was normalized by control (absence of PME‐1) as 100%. *N* = 3 *: *P* < 0.05 vs control. The results are expressed as the means ± SE. Comparisons between the groups were performed by one‐way analysis of variance, followed by Student–Newman–Keuls test. (D) Recombinant His‐PME‐1 WT, S156A, and M335D mutants were incubated with PME‐1 KO MEFs cell extracts. His‐PME‐1 was pulled down with Ni‐NTA agarose, and association of His‐PME‐1 with endogenous PP2Ac was analyzed by immunoblotting. Representative images from three independent experiments are shown. (E) Recombinant GST‐PME‐1 WT, S156A, and M335D mutants were incubated with recombinant His‐PP2Ac with 5 mm EDTA, 10 mm MgCl_2_, or 0.2 mm MnCl_2_. GST‐PME‐1 was pulled down with Glutathione Sepharose beads, and association of GST‐PME‐1 with His‐PP2Ac was analyzed by immunoblotting. Representative images from three independent experiments are shown.

Based on the cocrystal structure 14, PME‐1 associates with PP2Ac at two regions: the active site pocket and the PP2A binding interface (Fig. 3A). The six carboxyl‐terminal amino acids of PP2Ac are bound in the active site pocket of PME‐1, and a separate PP2A binding interface of PME‐1 binds at the active site of PP2Ac 12, 14. The fact that ABL127, but not OA, efficiently blocked PP2Ac demethylation suggests that blocking PME‐1 association with the active site of PP2Ac is not sufficient to inhibit PP2Ac demethylation. To clarify this point, we added ABL127 or OA in the cell extracts and analyzed the effects on the PME‐1/PP2Ac association using the His‐PME‐1 pull‐down assay. The PME‐1 KO MEFs were lysed in the standard buffer, and the cell extracts were incubated with DMSO, ABL127 (at 0.1 and 1 μm), or OA (at 10 and 100 nm). We found that addition of either ABL127 or OA reduced the association of PP2Ac with PME‐1 in a dose‐dependent manner (Fig. 3B), suggesting that both the active site pocket and PP2A binding interface have a role in PME‐1/PP2Ac association.

We further examined whether stable association of PME‐1 is inevitable for PP2Ac demethylation using recombinant PME‐1 mutants: S156A and M335D. Ser156 is essential catalytic residue of PME‐1, and the S156A mutation renders PME‐1 inactive. Based on the cocrystal structure, Met335 of PME‐1 is involved in H‐bond formation with PP2Ac 14 and has been suggested to inactivate PP2Ac by evicting catalytic metal ions from PP2Ac. We analyzed the effects of PME‐1 mutants on PP2A activity by *in vitro* PP2A activity assay. We found that PME‐1 WT and S156A significantly suppressed PP2A activity, while M335D mutant did not (Fig. 3C). We analyzed PP2A binding with PME‐1 mutants by pull‐down assay using recombinant His‐PME‐1 and PME‐1 KO cell extracts. Association of PP2Ac with the PME‐1 WT and the S156A mutant was confirmed, as in our previous report (Fig. 3D) 13. In contrast, the PME‐1 M335D mutant bound substantially less PP2Ac. Free metal ions in the assay buffer are necessary to analyze PP2A activity. We also examined PP2A binding with PME‐1 mutants in the buffer with/without metal ions by GST pull‐down assay using recombinant GST‐PME‐1 mutants and His‐PP2Ac (Fig. 3E). We found that the PME‐1 WT and the S156A mutants, but not the M335D mutant, associate with recombinant His‐PP2Ac, and these associations were not affected by the presence of free metal ions in the assay buffer.

### A stable association of PME‐1 with PP2Ac is not required for PP2Ac demethylation

**Figure 4:**
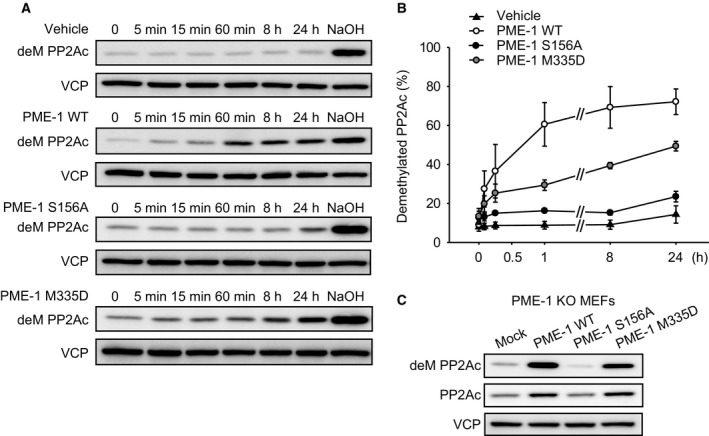
A stable association of PME‐1 with PP2Ac is not required for PP2Ac demethylation. (A,B) Cell extracts of PME‐1 KO MEFs were incubated at 30 °C for indicated time periods with recombinant His‐PME‐1 WT, S156A, and M335D mutants. Demethylation of PP2Ac was analyzed by immunoblotting with anti‐demethylated PP2Ac antibody (deM PP2Ac), and the ratio of deM PP2Ac was determined by NaOH‐treated cell extracts as 100%. Representative images (A) and quantitative data (B) from three independent experiments are shown. The results are expressed as the means ± SE. Comparisons between the groups were performed by one‐way analysis of variance, followed by Student–Newman–Keuls test. (C) Cell extracts from the PME‐1 KO MEFs expressing FLAG‐PME‐1 WT, S156A, and M335D mutants were analyzed by immunoblotting using anti‐deM PP2Ac antibody and total PP2Ac antibody. Representative images from three independent experiments are shown.

We examined demethylation activity of these PME‐1 mutants by replenishing PME‐1 KO cell extracts with recombinant PME‐1. This assay took advantage of the absence of PME‐1 activity in these extracts (see Fig. 2D) while using the endogenous PP2A as substrate. The PME‐1 M335D mutant catalyzed PP2As demethylation although the efficacy was lower by about half compared to PME‐1 WT (Fig. 4A,B). We further confirmed the methyl‐esterase activity of PME‐1 M335D in live cells by expressing the PME‐1 mutants in PME‐1 KO MEFs. Cell extracts from the MEFs were immediately (without incubation) taken for immunoblotting. The PME‐1 KO MEFs expressing FLAG‐PME‐1 WT or M335D, but not PME‐1 S156A, showed much lower PP2Ac methylation levels (Fig. 4C). These results suggested that PME‐1 reacts with PP2Ac for demethylation, even without forming a complex that can be recovered by pull‐down assay.

### PP2Ac association with various B subunits is differentially affected by PME‐1 methyl‐esterase inhibitor

**Figure 5:**
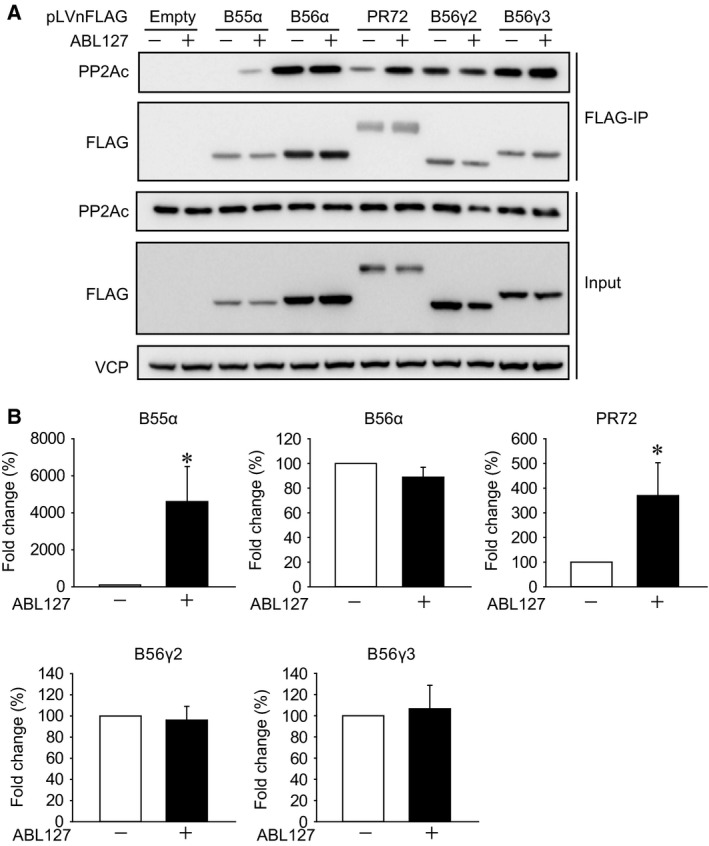
PP2Ac association with various B subunits is differentially affected by PME‐1 methyl‐esterase inhibitor. (A,B) FLAG‐B55α, FLAG‐B56α, FLAG‐PR72, FLAG‐B56γ2, and FLAG‐B56γ3 were transiently expressed in HEK293T cells. Cells were lysed in a buffer with or without ABL127 (1 μm), FLAG‐B subunits were immunoprecipitated with FLAG‐M2 beads, and the association with endogenous PP2Ac was analyzed by immunoblotting. Representative images (A) and quantitative data (B) are shown. The results are expressed as the means ± SE. Comparisons between the groups were performed by one‐way analysis of variance, followed by Student–Newman–Keuls test. *N* = 4 (B55α), 7 (B56α), 6 (PR72), 4 (B56γ2), and 4 (B56γ3). *: *P* < 0.05 vs without ABL127.

Immunoprecipitation is widely used to detect the association of PP2Ac with the B subunits. Here, we show data that PP2Ac association with a subset of B subunits is affected by PP2Ac demethylation in extracts. We analyzed the effects of ABL127 on the PP2Ac association with various B subunits. FLAG‐tagged B subunits were transiently expressed in HEK293T cells, and the cells were lysed in CHAPS buffer with or without added ABL127. The cell extracts were incubated with FLAG‐M2 beads for 3 h, and their association with the endogenous PP2Ac was analyzed by immunoblotting. We found that the association of PP2Ac with B55α and PR72 was enhanced many fold by ABL127 (Fig. 5A,B). On the other hand, PP2Ac association with B56α, B56γ2, and B56γ3 was not affected by ABL127. Together with the results in Figs 1 and 2, the data indicate that PP2A trimers with B56 subunits are insensitive to changes in PP2Ac methylation, whereas B55 and PR72 preferentially associate with methylated PP2Ac. PME‐1 methyl‐esterase inhibitor in the cell extract is essential to precisely detect the association of PP2Ac with a subset of B subunits through immunoprecipitation.

## Discussion

In a previous report, 100 nm of OA was shown to effectively inhibit the demethylation of PP2Ac *in vitro* 12. However, in the present study, addition of OA is not enough to effectively inhibit PP2Ac demethylation in cell extracts, especially at 30 °C. Consistent with our data, OA did not inhibit the demethylation of recombinant PP2Ac by recombinant PME‐1 25. The mechanism underlying this difference is still unclear. One possibility could be the difference of the assay system: We have used the whole‐cell extract, while the previous study used immunoprecipitated PP2A and extract from bacteria expressing human PME‐1.

Addition of OA in the buffer to prevent the demethylation of PP2Ac is based on the idea that OA fills the active site pocket of PP2Ac, hence preventing the access of PME‐1 by overlapping the binding interface 21. Structural study has suggested that PME‐1 interacts with the active site of PP2Ac and displaces the metal ions from there, hence stabilizing the PME‐1/PP2Ac association 14. We found that a mutation of Met335 to aspartate renders PME‐1 reduced binding to PP2Ac. The molecular mechanism for this remains elusive. Based on the structural study, residue Met335 of PME‐1 induces removal of catalytic metal ions from the PP2Ac active site due to the hydrophobic nature of the methionine residue that disfavors metal binding at the active site 14. PP2A inactivation step by PME‐1 follows a slow kinetics because it takes time to evict metal ions. Therefore, it is possible that PME‐1/PP2Ac interaction is detectable during the metal ion removal step, and the M335D mutant has reduced binding to PP2Ac because it does not evict metal ion. Structural study also suggested that Met335 is involved in H‐bond formation with PP2Ac. Although PP2Ac associates with the main chain of Met335, M335D mutation may affect relative localization of helix structure of main chain through the change of the side chain position. Therefore, low binding ability of PME‐1 M335D may be caused by loss of H‐bonds between PME‐1 and PP2Ac.

Our data, using the recombinant PME‐1 mutants, showed that reduced PME‐1/PP2Ac interaction led to a delayed catalytic reaction, but did not completely stop PP2Ac demethylation. PP2Ac demethylation was also observed in the cells; PME‐1 KO MEFs expressing PME‐1 WT and M335D showed lower PP2Ac methylation levels. Consistent with our previous reports 13, PME‐1 WT, but not S156A, increased the PP2Ac protein levels. In the present study, we observed that PME‐1 M335D also increased the PME‐1 protein levels, confirming that PME‐1 M335D retains the methyl‐esterase activity. These results indicated that the inhibition of PME‐1/PP2A association at the PP2Ac active site by OA is not sufficient to prevent PP2Ac demethylation.

Evidence from multiple studies indicates that the methylation of PP2Ac plays an important role in its association with a subset of B subunits, especially the B55 family 9, 10, 13, 26, 27. In the present study, the inhibition of PP2Ac demethylation by ABL127 during immunoprecipitation enhanced recovery of PP2Ac with B55α or PR72. These results support the idea that PP2Ac demethylation in cells or in cell extracts leads to dissociation of PP2Ac/B55 and PP2Ac/PR72 complexes. On the other hand, several reports show that the *in vitro* assembly of a PP2A holoenzyme between the PP2A AC dimer and B55 subunit does not require PP2Ac methylation 25, 28 Recent study shows that the TOR signaling pathway regulator (TIPRL) selectively induces disassembly of unmethylated PP2A holoenzyme 29. The methylation of PP2Ac hinders TIPRL binding and thus weakens TIPRL's ability to attack the PP2A holoenzyme. It is possible that PP2Ac methylation is not essential for the assembly of a B55‐containing PP2A holoenzyme, but it may stabilize the trimer. Further investigation will clarify the regulatory mechanisms for PP2A holoenzyme assembly.

It has been reported from GST pull‐down experiments using HA‐tagged PP2Ac mutants that PR72 does not absolutely require methylated PP2Ac for the holoenzyme assembly 26. Because the association of PP2Ac and PR72 was detectable even without ABL127, it is possible that PR72 could form a holoenzyme with demethylated PP2Ac. Regardless, our results show the importance of adding a PME‐1 methyl‐esterase inhibitor in the buffer for PP2A immunoprecipitation. On the other hand, association of PP2Ac with the B56 family was not dependent on or affected by the presence of ABL127, supporting previous proposals that PP2Ac methylation has little influence on B56 binding 30, 31, 32, 33. Another possibility could be that B56‐containing PP2A holoenzyme is resistant to PME‐1‐mediated demethylation. Our observation that demethylation levels of PP2Ac reached a plateau and did not reach 100% is in line with the possibility of PME‐1 resistant PP2A complexes.

## Author contributions

TO and RY designed the research; RY, ST, SM, and TI performed the research; TU and KS provided suggestions and discussion; TO and RY wrote the manuscript.
